# An Ishmaelite

**Published:** 1888-07-28

**Authors:** Leslie Keith

**Affiliations:** Author of "The Chilcotes," "Uncle Bob's Niece," "East and West," etc.


### An Ishmaelite.

By Leslie Keith,

Author of "The Chilcotes," "Uncle Bob's Niece," "East and West," etc.

*(Continued from p. 266.)*

### Chapter XVI.—On the Track.

The cripple, George Tucker, however, was not of Barbara's mind.

He began think it was his business—more than that, his mission—to find out all that he could about the mysterious young man who had been suddenly added to the family circle. His discoveries were of course to be adverse to the stranger's comfort, and disastrous to his continued stay under the Proudie roof.

He never ventured out, he had no friends who came to see him, except the cousin who was probably no better than himself; no letters or news from the outer world ever reached him. These facts were surely significant enough.

Tucker assured himself over and over again that you couldn't throw dust in his eyes. "He ain't here for nothink," he said to himself in those moments when he entertained his conjectures. "'E's no more been brought up to the libery business than I've been brought up to be a markiss. 'E knows a deal too much, 'e do."

It was not the quantity of his knowledge that alone marked Fred off from "one of us" in Mr. Tucker's eyes, it was the quality as well. Even the very little Mr. Eastgate had been heard to say in the other's presence was quite sufficient to justify his suspicions of Fred's superiority. There is a way of putting your sentences, even if they are only six words long, that you can't acquire unless you have had "tip-top" advantages. Having, by his own unconscious proving, had "tip-top" advantages (to quote Tucker), on what grounds was Eastgate hiding those lights under a Bermondsey bushel? Was it for the love of Delia? The most wakeful jealousy scarcely allowed the suspicion. Perhaps it was Barbara for whom he exiled himself; perhaps it was for some much darker reason. Whatever the cause, Tucker meant to find it out.

The easiest way to accomplish this seemed to be to follow Arthur, and to find him out. This, however, was not so simple a matter as it seems. In the first place, Arthur was an active young fellow, whereas Tucker was handicapped by the necessity for using a crutch. Then Arthur's visits were made at uncertain and eccentric times, often when the exigencies of the eating-house demanded Tucker's presence there. Even when Arthur came in the late evening, and Tucker's leisure allowed him to follow his departing steps, and track him through one or two streets, Arthur frequently only waited till he reached a main thoroughfare to hail a passing cab and be whirled off under his angry pursuer's eyes.

The cripple had no money to throw away on hansoms, in which to continue the chase; the study of the Directory helped him scarcely more. There were several Eastgates mentioned in it, of various occupations, none of which seemed quite to fit the handsome young man who had so much leisure to give to Gillespie Street.

It is usually an easy enough thing to find out all one wants to know about anyone who has a club or a private address, and a recognised position in society. But in saying this, one does not look at the matter from a George Tucker's point of view. South London and West London are two nations, living side by side, and yet scarcely knowing anything of each other. Tucker's acquaintance with fashionable London was of the slenderest. His infirmities kept him from the pleasures of socialistic demonstration, and his life since, as a charity schoolboy, John Proudie had taken compassion on him, had been mainly passed in the district that includes Kennington, Walworth, and Bermondsey in its area; and even if he had heard of Fred's fall, six years might well blunt the keenest memory for such details.

For a long time, then, Tucker's schemes were thwarted by divers hindrances, but at last fortune favoured him.

Arthur happened to come to Gillespie Street once upon a Sunday morning, when all the household excepting the two assistants were absent. John Proudie was a regular attendant at a neighbouring chapel, where his daughters sang in the choir, Delia's voice ringing out clear above the others with an untrained, natural sweetness. The cripple sometimes went to stare at her, and listen to her, but on this particular Sunday he preferred to remain at home and watch Fred, as if by looking at him hard enough he could surprise his secret.

Fred, aware of the scrutiny and coldly disliking it, took a book from the shelves and sat down as far from his companion as the narrow dimensions of the room allowed. Tucker getting nothing but monosyllables in reply to his remarks, relapsed at last into sulky silence, and it was thus Arthur found the ill-matched pair.

The tinkle of the bell gave warning of his entrance, and Fred looked up. He had ceased to remonstrate with his cousin on the score of his visits, and perhaps on this special day he would have been sorry if Arthur had taken him at his word. Arthur nodded to Tucker and offered him a cigar, which was accepted ungraciously.

"Fred," said Arthur, "come out. There's scarcely a soul about, and the sun is shining."

Fred shrank back. "I haven't been out since ——"

"I know," said Arthur. "That's the best reason for

* "A Manual of the Diseases of India, with a Compendium of Diseases Generally." By W. J. Moore, C.I.E., Surgeon-General with the Government of Bombay, Honorary Surgeon to the Viceroy of India, &c. Second Edition. London: J. and A. Churchill.

* The Holy Bible, containing the Old and New Testaments, to which are appended: Notes Analytical, Chronological, Historical, and Geographical; A Biblical Index; Concordance; Dictionary of Scripture Proper Names; a Series of Maps; and a Compendium of Scriptu.e Natural History.

coming now. You can't shut yourself up for the rest of your life, and even the air of Gillespie Street is better than none. Come along; I want to talk to you."

He glanced round at the cripple as he spoke, and Fred's eyes followed the same direction. Perhaps it was the conviction that nothing short of forcible ejection would rid them of Tucker's presence that helped his wavering decision. He got up and left the room, coming back presently with his hat.

Arthur passed an arm through his cousin's when they stepped upon the pavement, the door of the shop shutting with a tinkle behind them.

"Does that little beast always distinguish you with his company?" he asked. "I never miss seeing him, whoever else may be absent."

"He's rather ill conditioned, and he seems to have a special spite at me. Sometimes I think he knows ——"

"Nonsense," said Arthur strongly, "he can't know.' Then he added illogically, "Even if he does, I dare say he has done a thousandfold worse himself."

"Two blacks won't make a white," said Fred sententiously; "and still less can the pot afford to call the kettle black ——"

"Look here," said Arthur, who seemed to be rather depressed and out of spirits, "I didn't come here for you to spout proverbial philosophy at me. Are you coming for a day in the country—on the river—where you will—with me?"

"Do you think it likely? The river!"

"Well, it needn't be the river—somewhere, anywhere where its clean——"

"It is clean enough for me here. Go yourself, Arthur; I'm sick of telling you you have no business here."

"You are my business here. If you want to explore Bermondsey, in Bermondsey we'll stay."

They strayed at last into a churchyard, long since disused, but kept with a certain decency and order. The grass about the mounds was roughly cut, and the grey stones, falling aslant as if wearied by their watch over the sleepers beneath, were railed in from desecration. A thin flutter of leaves from the stunted trees that lined the main walk—early touched with autumn—fell silently, scarcely drifting in the still air.

The churchyard made an open space among the crowding roofs in which the parish church stood with an air of aloofness. It had no architectural beauty, but it carried a soothing sense of repose and peace to the two, who found a flat, unrailed stone and seated themselves on it; all the sounds that reached them were pleasant ones. The Sunday traffic here fell off and was hushed, and on the silence there rose now and then a chanted amen from the worshippers, or the deep *Bourdon* notes of the organ. Something in the place and its associations kept them from speech for a little, and neither of them guessed that behind a neighbouring tomb a listener was lying in wait for their words.

Fred spoke at last.

"Arthur," he said, "I had a letter from Nellie ——"

Arthur looked up with a sudden brightening of his downcast face. "From Nellie? That's good ——"

"No, it is all wrong. Listen to me. If Nellie proposes to come here—and I'm afraid she may—you must forbid her."

"Why?"

"You know why as well as I. If Nellie had been free, and if in the kindness of her heart she had wished me to feel that she forgave me, we might have met again, painful as it would have been for both of us. But Nellie has her child, and her husband, and her friends to think of. Her husband would scarcely care to let her come here."

"Bellynge is an ass!" said Arthur testily.

"No; he is a wise man if he forbids Nellie to have anything to do with me. You can't look on those things with my eyes, Arthur. You haven't had five years of exile to do your thinking in."

"Thank heaven I can't!" said Arthur fiercely. "If I were as morbid and, desponding as you I should hang myself, or drown myself, and have done with it."

"Do you think I've never been tempted that way?" said Fred sadly. "I haven't left myself much of a life, have I? I've fallen, Arthur, and I'm not of the stuff to rise again. A man who was braver and less weak might have done it, but not I."

"We all may be conquerors of our fate if we will! If one fatal step is to settle the whole business for us——"

"When you've been down in the mire, you can't wash out the stain. I've repented, I hope, if repentance means willingness to dree one's weird. Let me be, Arthur. I've a thousandfold better refuge than I've deserved, and while I hurt nobody I will stay in it. But keep Nellie away. Tell her, if you like, that I burnt her letter, and that I am dead to her."

Arthur made no answer. He was dull and out of spirits, and Fred's words had a ring of hopelessness in them that further depressed his mood. The leaves fluttered down upon the dead who had given up the battle, and from the church hard by there rose a murmur of voices lifted in unison. What was it they were saying?

"I believe in the resurrection of the dead, and in the life of the world to come"—the life of new chances—the world that "sets this right."

"Fred," he said, after a long time, when, perhaps, he too, had repeated his *credo*, and taken hope from it, "what made you do it?"

Fred drew himself up, and looked straight before him at the dusky blue of the city sky. The blood rushed up into his haggard face, but he spoke quite quietly.

"I was in despair," he said, "and I couldn't bear to see my mother's tortured looks. I hid myself for a day, but there was nothing but ruin and disgrace before me. Then I thought of old Mack—there was a chance that he would listen and help me. And I went to his house and heard that he had been suddenly called from home. But the housekeeper knew me, and let me go into the library to write to him. I was desperate; if there had been money within reach I would have taken it. You know what a careless old fellow he is; there was no money, but his cheque-book lay there on his desk. The devil entered into me, and I signed myself over to him. I suppose, like other fools, I hoped some miracle would happen to save me from the consequences of my act. You know the rest. Come, let us get up. The people who omit the sermon will be coming out of church presently, and it is time for you to get back to Portland Place."

The listener behind the tombstone rose from his cramped position, and followed them at a safe distance. Very little of what had passed between the cousins had reached him, but that little was enough. "Portland Place." He made a note of that and of other phrases he had caught, and that fitted into his theory like the pieces of a puzzle.

"I will walk with you to the door," Arthur said. And as they neared it, the little family party, released from the chapel, advanced to meet them.

Delia and Barbara came first. The younger girl's blushes deepened when she saw the cousins coming towards them arm-in-arm. The sisters were dressed alike, in a quaker-like grey that enhanced the younger's beauty, and suited well with the elder's quiet, grave looks. The honest Proudie walked behind in his shiny, Sunday black, with his wife, wearing a yellow shawl, hanging on one arm, and Miss Betsy grimly in possession of the other.

There was no mistaking the class to which they belonged. But it flashed across Arthur as he looked at the sisters that, in spite of their social inexperience, they might have moved in any circle with distinction. It is often so with girls whose fathers and brothers remain at the old barbaric level, but it is not always that this feeling for greater refinement and culture leaves the affections loyal and unshaken. But when Delia lovingly draped the yellow shawl round her mother's shapelesss shoulders, and when Barbara brushed her father's glossy Sunday hat, neither of them, be sure, would have changed those good, honest, vulgar folks for genteeler parents if a wish could have done it.

When the groups met, a great deal of hearty handshaking ensued, and Arthur received a cordial invitation to share the family joint in the room where he had broken his fast more than once.

He hesitated, he glanced at Delia; but before he could reply, Fred, who was usually so dumb, struck in with—

"Miss Coates has come home, hasn't she? She will expect you to lunch." Delia looked at him wonderingly, and Arthur looked at him too, but with some resentment and reproach. Was Fred turning mentor?

"Yes," he said, "she got back last night. I suppose I must go."

"Miss Coates," whispered the cripple—who had joined the group—in Delia's ear, "I bet you anything she's his sweetheart."

Delia turned a quivering, indignant face upon him.

"Well, and why not?" she demanded; "why should there not be a lady who loves him?"

"Oh, if it don't bother you, it don't bother me," said Tucker, with a sneer.

*(To be continued.)*

